# Dataset on digital technologies as learning content in farm manager training in Switzerland and willingness to use farm information systems

**DOI:** 10.1016/j.dib.2023.109113

**Published:** 2023-04-05

**Authors:** Jeanine Ammann, Achim Walter, Nadja El Benni

**Affiliations:** a Agroscope, Research Group Economic Modelling and Policy Analysis, Ettenhausen, Switzerland; b ETH Zürich, Institute of Agricultural Sciences, Zürich, Switzerland; c Agroscope, Research Division on Sustainability Assessment and Agricultural Management, Ettenhausen, Switzerland

**Keywords:** Teaching, Farm management, Farm information system, Digital technology, Smart farming

## Abstract

This article describes the data from an online survey conducted at a farm management course in Switzerland. The survey was conducted in German and French between April and May 2021. It was emailed to teachers and students at agricultural education centres across Switzerland that offer a farm management program. In the first part, the survey investigated whether digital technologies were taught in agricultural training, and, more specifically, in basic training or in the farm management course. Next, it investigated teachers’ and students’ general perceptions of digital technologies in plant production and animal husbandry. The survey further included questions about information sources individuals use to learn more about digital technologies in agriculture. In a subsequent part, students who already owned or co-owned a farm were asked whether they use a farm management information system and were planning to use more digital technologies in the future. For this, we used three items investigating perceived ease of use, which were derived from a previous study and four items using a trans-theoretical model of adoption. Finally, all participants provided basic sociodemographic data and answered items related to environmental concern, based on an existing scale. The survey can be used and adapted to different contents, aiming to investigate perception and adoption of farm management information systems and study the course content, how individuals acquire knowledge or how they perceive digital technologies.

**Specifications Table**

**Table:** 

Subject	Social Science

Specific subject area	Smart farming, teaching, farm management course
Type of data	CSV file
How the data were acquired	Data were acquired via an online survey, programmed on and conducted with an online survey tool, Unipark (Management Questback GmbH, Germany). E-mails were sent to all institutions that offer a farm management course in Switzerland, inviting teachers and students of the farm management course to participate. The survey was available to participants in German and French.
Data format	Raw, wide format
Description of data collection	Data were collected through an online survey sent to teachers and students of a farm management course in Switzerland. Data was collected between April and May 2021.
Data source location	Region: Europe Country: Switzerland
Data accessibility	The data is hosted on Zenodo (zenodo.org). Repository name: Online survey among students and teachers in the Swiss farm management course Data identification number: 7108132 Direct URL to data: https://zenodo.org/record/7108132 [1]
Related research article	Ammann, J., Walter, A., El Benni, N., Adoption and perception of farm management information systems by future Swiss farm managers – An online study, Journal of Rural Studies, 89, 298-305 (2022) https://doi.org/10.1016/j.jrurstud.2021.12.008. [2]

## Value of the Data

• The data describe the current educational situation in a farm management course and agricultural training, in general, in Switzerland. With that, the data can be used as baseline for future analyses.
• The data investigate future farm managers’ willingness to use farm management information systems and their current state of adopting technology.
• The data provide insights on the knowledge and needs of future farm managers in terms of digital technologies, in general, and specifically for farm management information systems (FMIS)
• Given that digital technologies in agriculture are a global and emerging topic, this data from Switzerland is relevant for other countries and future studies alike.
• The survey can be used and / or modified for use in other contexts or countries and thereby enable cross-cultural comparisons.
• The survey contains newly developed items measuring technology perception which can be further developed and validated in future studies.
• Analysing the survey design and / or the data obtained with it can serve pedagogical purposes (e.g., for statistics lectures) by providing real data that students can work with.

## 1 Data Description

The data was collected via an online survey in April and May 2021 that was emailed to teachers and students at agricultural education centres across Switzerland that offer the farm management program. The original datasets in wide format (CSV file and SPSS file); an English translation of the dataset in wide format (CSV file); origneiinal surveys in German and French (Betriebsleiter_survey_french.pdf and Betriebsleiter_survey_german.pdf); and a codebook in English (Betriebsleiter_Codebook_English.pdf) are available at the Zenodo repository:

https://zenodo.org/record/6901229

### 1.1 Experimental Design, Materials and Methods

Our survey was programmed conducted using the online survey tool, Unipark (Management Questback GmbH, Germany). The link to the survey was sent to the agricultural education centres across Switzerland that offer the farm management course. Teachers and students of the farm management course were invited to take part in this survey and complete it within two weeks. After these two weeks, one reminder was sent, reminding them to complete the survey and were given another week to do so. As a result, the data collection took in total three weeks in April and May 2021. Participants who indicated that they were neither teachers nor students in the farm management course were screened out. As an incentive for participation, we offered participants the possibility to receive a short summary of the results.

In total, 41 teachers and 109 students participated in the survey (Table 1). Those who indicated that they were neither students nor teachers (v_520 = 3, n = 6) were excluded. Of the 109 students, 23 indicated that they were already managing a farm. To ensure participant anonymity, information regarding their education centre was collected, but deleted after checking for spatial distribution of responses.

**Table 1:** Sample description (N = 150).

	N	French-speaking	Age			Female	Environmental concerns
		[%]	M	SD	Range	[%]	M (SD)
Teachers	41	20	43	13	23-61	24	5.0 (0.9)
Students	109	18	28	5	21-51	16	4.1 (1.1)

*Note.* Environmental concerns: averaged scale across four items according to [3], measured on a scale from 1 to 6.

The total survey duration was around 15-30 minutes. It was available in German and French. The Statistical Packages for Social Sciences (SPSS) version 26 and Microsoft Excel were used to code data and run statistical analyses.

The survey had the following parts:

1. Digital technologies during training [all participants]
  a. Taught or not taught
  b. Materials used in teaching
  c. Most important learning contents [*qualitative*]
2. Perception of digital technologies in plant production [all participants]
3. Perception of digital technologies in animal husbandry [all participants]
4. Information procurement [all participants]
5. Owns farm
  a. Family farm or not
  b. Production branch
6. Farm management information systems (FMIS)
  a. Taught or not taught
  b. Perception and use of FMIS [students with farms only]
7. Environmental concerns and sociodemographics [all participants]

**Fig. 1:**
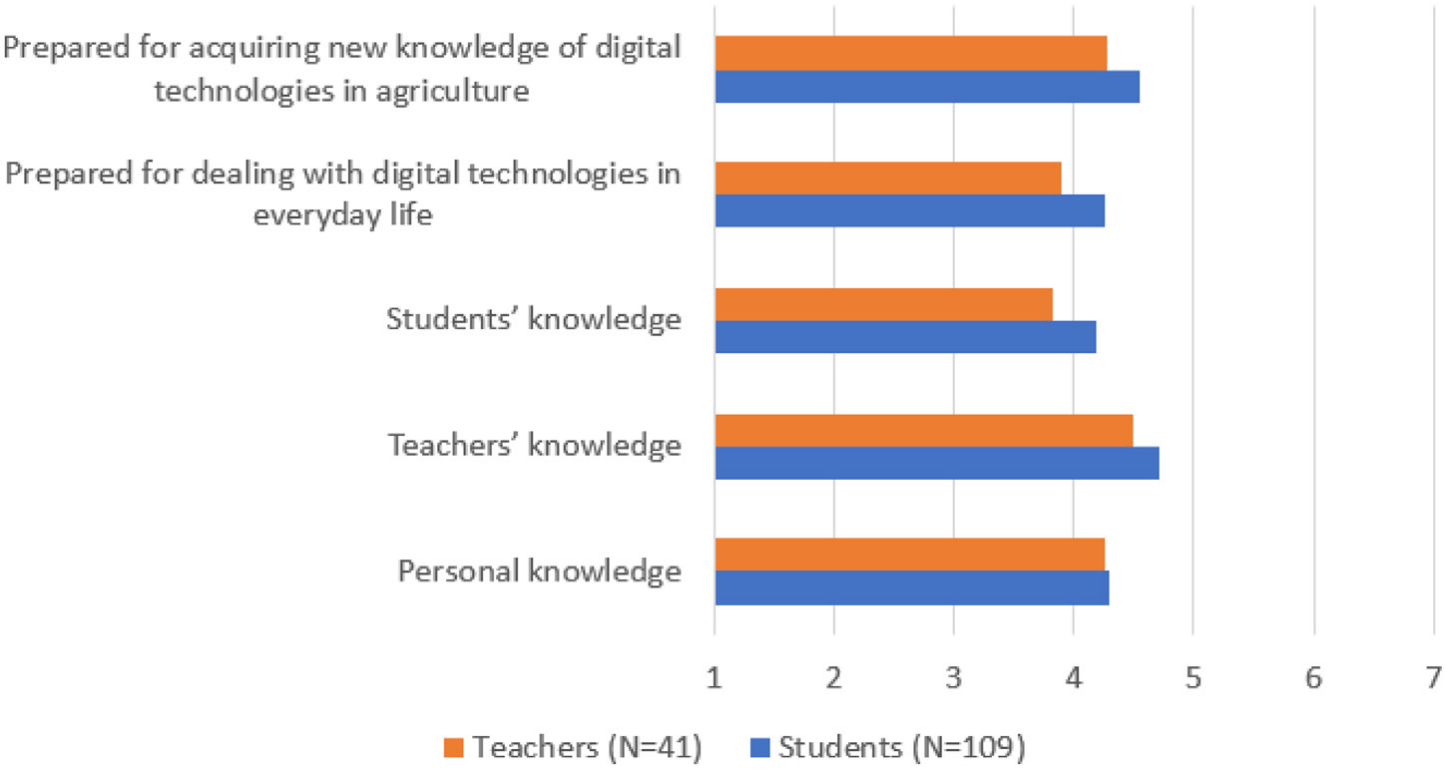
Digital technologies in agricultural education (part 1 of the survey).

Part 1 of the survey addressed whether digital technologies were taught during agricultural training. Further, it investigated how digital technologies were taught, what the most important learning contents in the course were (qualitative data) and how prepared the students felt to deal with digital technologies in everyday life (see Fig. 1). Students and teachers further estimated their personal and subjective level of knowledge as well as their knowledge of digital technologies, both measured as self-report on a scale from 1 (very little knowledge) to 7 (a lot of knowledge).

In parts 2 and 3 of the survey, the general perception of digital technologies in agriculture were investigated, using nine items for plant production and eight items for animal production (see Table 2). These items were originally developed by the authors for the context of vegetable farming and first tested in a previous study [4], where the data has been made available [5]. In the present study, the items were adapted to both a plant- and an animal-related context to adapt them to a broader context.

**Table 2:** Perception of digital technologies in agriculture (parts 2 and 3 of the survey, N = 150).

		Students N = 109			Teachers N = 41		
		Mode	M	SD	Mode	M	SD
**Plant Production**							
1	In the next 2 years, resources (pesticides, fuel, water, etc.) can be used in a more targeted manner, and thus saved through an increased use of digital technologies.	7	5.9	1.3	7	5.9	1.1
2	In the next 2 years, the labour time requirement (e.g. per kilogram of vegetables produced) will decrease due to the increased use of digital technologies.	5	5.0	1.4	5	4.8	1.5
3	In the next 2 years, problems such as soil compaction or humus depletion can be reduced through the increased use of digital technologies.	5	4.4	1.7	5	4.5	1.5
4	In the next 2 years, the wage costs for auxiliary staff will decrease due to the increased use of digital technologies.	5	4.7	1.6	4	4.2	1.6
5	There is a risk that increased use of digital technologies will lead to less consumer acceptance of the items produced.	4	3.4	1.6	2	2.9	1.7
6	Increased use of digital technologies leads to simplified recording, documentation and evaluation of the collected data on farms.	7	5.4	1.5	6^a^	5.3	1.6
7	Increased use of digital technologies promotes environmental protection.	4	4.9	1.5	5	5.0	1.5
8	Increased use of digital technologies facilitates plant health monitoring.	5	4.8	1.7	6	5.6	1.1
9	Increased use of digital technologies leads to less administrative work (e.g. when applying for direct payments).	5	4.3	1.7	4	4.8	1.2
**Animal production**							
1	In the next 2 years, the demand for labour time (e.g. per kilogramme of meat produced) will decrease due to increased use of digital technologies.	4^a^	4.3	1.5	5	4.2	1.3
2	In the next 2 years, the wage costs for auxiliary staff will decrease due to the increased use of digital technologies.	5	4.2	1.5	4	3.7	1.3
3	There is a risk that the increased use of digital technologies will lead to less consumer acceptance of the animal products created.	5	3.8	1.9	2	3.5	1.7
4	Increased use of digital technologies leads to a reduction in veterinary costs.	5^a^	4.8	1.6	5	4.6	1.2
5	Increased use of digital technologies leads to simplified recording, documentation and evaluation of the collected data on farms.	7	5.3	1.5	5	5.2	1.2
6	Increased use of digital technologies promotes environmental protection (e.g. less greenhouse gases).	4	4.0	1.7	4	3.9	1.4
7	Increased use of digital technologies facilitates animal health monitoring.	6	5.5	1.4	6^a^	5.7	0.8
8	Increased use of digital technologies leads to less administrative work (e.g. when applying for direct payments).	4	4.3	1.6	4	4.8	1.3

*Note.* Items were rated on a scale from 1 (do not agree at all) to 7 (agree completely). ^a^ Multiple modes exist, the smallest value is reported.

In part 4 of the survey, teachers and students were asked how they look for additional information on digital technologies; that is, what sources of information they use to inform themselves (Table 3). Using a given list of information sources, they rated each for its importance in their search of more information on digital technologies.

**Table 3:** Information procurement: how teachers and students inform themselves about digital technologies used in agriculture (part 4 of the survey, N = 150).

Information source	Teachers		Students	
	n = 41		n = 109	
	M	SD	M	SD
Agricultural trade newspapers/magazines	5.5	1.3	5.6	1.3
Own research on the internet	5.9	1.2	5.9	1.2
Professional colleagues	5.6	1.1	5.9	1.1
Teachers/school	5.1	1.4	5.1	1.4
Cantonal advice	4.3	1.6	4.4	1.6
Sales advice	4.6	1.6	4.7	1.6
Advisory centres	4.2	1.5	4.8	1.5
Research centres	4.7	1.8	4.9	1.8
Specialist conferences	5.3	1.6	5.3	1.6
Learning videos or video platforms	5.7	1.4	5.7	1.4

Note. Information sources were rated on a scale of 1 (not important at all) to 7 (very important) for their importance in the search for information about digital technologies.

In part 5 of the survey, information was obtained regarding students’ farms (Table 4). For some of the questions related to farm management, it was a prerequisite to know which students were already managing a farm and making their own decisions regarding what technologies to use. This group of students with a farm were filtered and directed to a set of questions regarding their farm. These questions were unavailable to teachers and students without a farm. Similarly, questions about the use of FMIS were only available to the subsample of students with a farm.

**Table 4:** Farm takeover and farm management (part 5 of the survey).

		[%]
Farm takeover (students, n = 109)		
	Taking over a farm in the family	73
	Taking over a farm outside the family	6
	Not clear yet	12
	Other	6
Farm management (students with farm, n = 86)		
	Managing the farm alone	57
	Co-managing the farm	37
	Not clear yet	6

Part 6 of the survey focused on farm management information systems. This part included three items investigating the perceived ease of use of FMIS, as derived from Michels et al. [6] and four items using a trans-theoretical model of adoption derived from Michels et al. [7].

## Ethics Statements

The researchers adhered to all ethical considerations during the data collection process and followed institutional [8] and psychological ethical guidelines [9]. All participants involved in the study provided their written, informed consent to participate. Participation was voluntary and could be withdrawn at any time. Participants remained anonymous and their responses were dealt with in confidence.

## CRediT authorship contribution statement

**Jeanine Ammann:** Conceptualization, Formal analysis, Investigation, Data curation, Writing – original draft. **Achim Walter:** Writing – review & editing. **Nadja El Benni:** Conceptualization, Writing – review & editing.

## Declaration of Competing Interest

The authors declare that they have no known competing financial interests or personal relationships that could have appeared to influence the work reported in this paper.

## Data Availability

Online survey among students and teachers in the Swiss farm management course (Original data) (Zenodo).

## References

[1] Ammann J., Walter A., El Benni N. (2021).

[2] Ammann J., Walter A., El Benni N. (2022). Adoption and perception of farm management information systems by future Swiss farm managers – an online study. J. Rural Stud..

[3] Shi J. (2016). Knowledge as a driver of public perceptions about climate change reassessed. Nat. Clim. Change.

[4] Ammann J., Umstätter C., El Benni N. (2022).

[5] Ammann J. (2022).

[6] Michels M. (2019). Entwicklung und validierung eines technologieakzeptanzmodells für die nutzung von forward-kontrakten in der landwirtschaft. Aust. J. Agric. Econ. Rural Stud..

[7] Michels M., von Hobe C.F., Musshoff O. (2020). A trans-theoretical model for the adoption of drones by large-scale German farmers. J. Rural Stud..

[8] Agroscope, Richtlinien für die wissenschaftliche Integrität und gute wissenschaftliche Praxis (Guidelines for scientific integrity and good scientific practice), Agroscope, Editor. 2019. https://www.agroscope.admin.ch/dam/agroscope/de/dokumente/ueber-uns/agroscope/wissenschaftliche-integritaet.pdf.download.pdf/richtlinien-wissenschaftliche-integritaet-de.pdf.

[9] Association A.P. (2002). Ethical principles of psychologists and code of conduct. Am. Psychol..

